# Pictorial Representation of Illness and Self Measure Revised II (PRISM-RII) – a novel method to assess perceived burden of illness in diabetes patients

**DOI:** 10.1186/1477-7525-6-104

**Published:** 2008-11-27

**Authors:** Sandor Klis, Ad JJM Vingerhoets, Maartje de Wit, Noortje Zandbelt, Frank J Snoek

**Affiliations:** 1 Clinical Psychology Section, Tilburg University, Warandelaan 2, Tilburg, The Netherlands; 2 Department of Medical Psychology, VU University Medical Center, Van der Boechorststraat 7, Amsterdam, The Netherlands

## Abstract

**Background:**

The Pictorial Representation of Illness and Self Measure (PRISM) has been introduced as a visual measure of suffering. We explored the validity of a revised version, the PRISM-RII, in diabetes patients as part of the annual review.

**Methods:**

Participants were 308 adult outpatients with either type 1 or type 2 diabetes. Measures: (1) the PRISM-RII, yielding Self-Illness Separation (SIS) and Illness Perception Measure (IPM); (2) the Problem Areas in Diabetes (PAID) scale, a measure of diabetes-related distress; (3) the WHO-5 Well-Being Index; (4) and a validation question on suffering (SQ). In addition, patients' complication status, comorbidity and glycemic control values(HbA1c) were recorded.

**Results:**

Patients with complications did have marginally significant higher scores on IPM, compared to patients without complications. Type 2 patients had higher IPM scores than Type 1 patients. SIS and IPM showed low intercorrelation (*r *= -.25; *p *< .01). Convergent validity of PRISM-RII was demonstrated by significant correlations between IPM and PAID (*r *= 0.50; *p *< 0.01), WHO-5 (*r *= -.26; *p *< 0.01) and SQ (*r *= 0.36; *p *< 0.01). SIS showed only significant correlations with PAID (*r *= -0.28; *p *< 0.01) and SQ (*r *= -0.22; *p *< 0.01). Neither IPM nor SIS was significantly associated with HbA1c. The PRISM-RII appeared easy to use and facilitated discussion with care providers on coping with the burden of diabetes.

**Conclusion:**

PRISM-RII appears a promising additional tool to assess the psychological burden of diabetes.

## Background

Living with a chronic disease like diabetes requires considerable psychological adjustment. Failure to adjust adequately to the disease may have negative consequences for the patient's quality of life, possibly resulting in suffering. An important outcome of this adjustment is the amount of suffering that a disease potentially causes. Although suffering is frequently mentioned in the medical literature, its definition and characteristics are often implied rather than defined. One definition that has been frequently cited is that of Cassell [1]: 'a state of severe distress associated with events that threaten the intactness of the person'. From this definition, it follows that the extent of suffering is not merely determined by the severity of the illness itself, but rather by the perceived threat it poses to the intactness of the self, i.e. the impact and meaning a disease has for a patient. In addition, personality factors are assumed to play an important role in coping with the illness [2].

The Pictorial Representation of Illness and Self Measure (PRISM), has recently been introduced as a generic measure of suffering [3]. This 'circle test' consists of a rectangular sheet of paper, with a yellow disk in the bottom right corner. Patients are instructed to imagine that the sheet represents their life, and the yellow disk their self, and to place a red disk which represents their illness, somewhere on the sheet, to reflect the position of the illness in their life. The distance between the centers of the two disks is labelled the Self-Illness Separation (SIS). Following Pincus and Morley [4], a separation between illness and self schema is assumed to signify a healthy adjustment to the illness.

The PRISM, assessing the subjective position of one's illness in relation to the self, seems to match this definition rather well. Indeed, a study among lupus patients found support for this 'enmeshment hypothesis' [5]. However, as the PRISM is a rather abstract instrument, and no golden standard measure of suffering exists, its validation yields serious problems. Nevertheless, studies in patients with rheumatoid arthritis [6], chronic obstructive pulmonary disease [7], systemic lupus erythematosus [8], psoriasis [9], vitiligo [10], and chronic pain [11] have provided evidence suggesting that PRISM measures aspects of suffering, and a validation study with over 700 patients from different disease groups showed PRISM to have good test-retest reliability, and to be sensitive to therapeutic change [12]. In addition, the PRISM has been found useful in differentiating alcohol-related disorders [13], and in assessing post-traumatic growth in bereaved parents [14]. Because the measure is generic, it allows for comparison of suffering not only across different diseases, but even including problems of a different nature. In a recent pilot study, using a modified version of the PRISM, substantial differences in SIS were found between different diseases, with breast cancer and lung disease patients reporting higher SIS than whiplash and infertility patients [15].

In the current study we aimed to assess whether a modification of the original PRISM task, the PRISM-RII [15] is a feasible and valid instrument for measuring suffering in people with diabetes. Diabetes was chosen, since this condition is a highly prevalent chronic disease that is known to cause considerable distress, related both to the symptoms and complications of the disease as well as the daily demands of self-management [16-18].

Much similar as in the Wouters et al. study [15], in the present study the original PRISM was also modified in three ways, without changing the conceptualisation of the measure. First, the single red illness-disk was replaced by three different sized illness disks (respectively smaller than, equal to and larger than the self disk), from which respondents were asked to choose one. This yielded an additional variable, the Illness Perception Measure (IPM), operationalized as the size of the chosen disk. IPM is hypothesized to measure the perceived severity of the illness. In addition, as a second variable SIS was computed measuring the perceived position of the illness in the patient's life. A second revision implied that the yellow self-disk was moved to the middle of the sheet, in the center of a large printed circle. This was done to make the visual analogy of the relative positions of illness and self more intuitive; it is easier to envisage that the self is located in the centre of one's life than in the bottom right corner. In addition, in our experience with the original PRISM, several patients put the illness disk in the center of the sheet, commenting that their disease took a central position in their lives. Finally, the PRISM-RII was administered computer-based.

If the PRISM-RII is a measure of diabetes-related suffering, the following predictions should be confirmed. First, as the amount of suffering is likely to be moderated by disease status, patients with diabetes related complications or comorbid disorders might be hypothesized to report more intrusiveness compared to those without complications or comorbid disorders, which might be reflected in a smaller IPM and higher SIS values. Second, SIS and IPM should be moderately intercorrelated, without suggesting redundancy. Finally, IPM and SIS are expected to be moderately related with measures of well-being and diabetes related distress, confirming convergent validity.

## Methods

### Patient sample

Three-hundred and eight diabetes patients participated in this study. The whole sample had a mean age of 50.7 years (SD = 16.9, range 19–89). Both sexes were equally represented. One-hundred and nineteen patients were diagnosed with type 1 diabetes. These patients had a mean age of 43.6 years (SD = 14.4, range 19–82). One-hundred eighty-nine patients were diagnosed with type 2 diabetes. These patients had a mean age of 59.8 years (SD = 13.1, range 28–89). Of the whole sample, 46% had at least one diabetes-related complication. Patients completed the questionnaires as part of the annual review at the Diabetes Outpatient Clinic of the VU University Medical Centre (VUMC) in Amsterdam, the Netherlands [19]. The study was approved by the Medical Ethical committee of the VUMC, and all participants gave written permission to use their (anonymous) data for scientific purposes.

### Measures

#### The Pictorial Representation of Illness and Self Measure Revised II (PRISM-RII)

**Figure 1:**
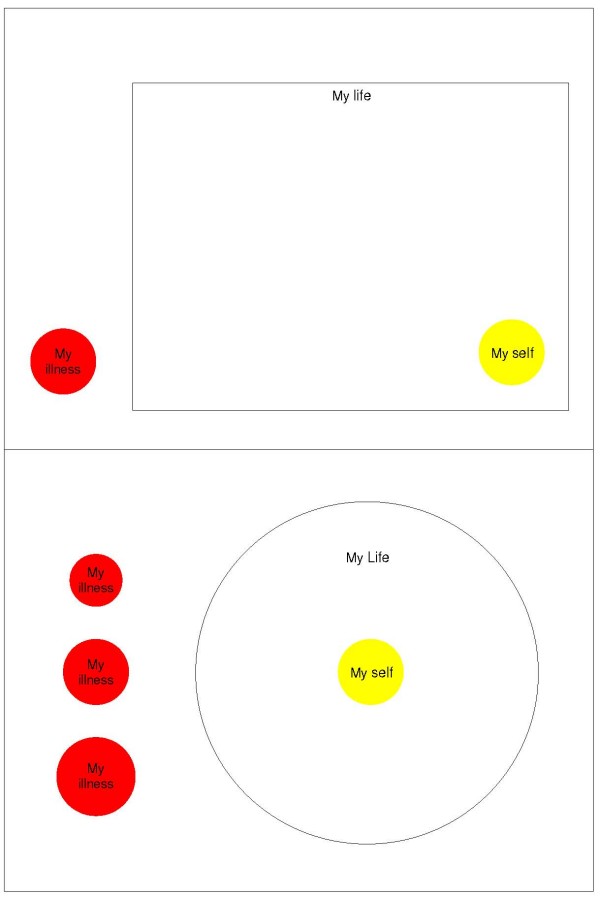
The PRISM (top) and the PRISM-RII (bottom).

The PRISM-RII [15], consists of a large white circle (186 mm in diameter), representing the respondents' life, with a yellow disk (52 mm in diameter) placed in the middle and in front of the white disk, representing the respondents' self (Figure 1). Three differently sized red disks were shown on the left side of the circle, representing the respondents' illness. The illness disks were respectively smaller than, equal to, and larger than the self disk (35 mm, 52 mm, and 65 mm in diameter). Patients were given the following written instruction: 'The white circle represents your current life and the yellow disk represents you. The three red disks represent your diabetes. Select from the three red disks the one which, in your view, represents your diabetes most accurately. Using the mouse, drag this disk into your life. Locate the disk at the place that the diabetes occupies in your life. You can place the disk anywhere in your life, also entirely or partially on top of your self.' Two measures were extracted from the PRISM-RII: Self Illness separation (SIS) in pixels, ranging from 0 to 300, and the Illness Perception Measure, ranging from 1 to 3, with 1 representing the smallest disk.

#### Problem Areas in Diabetes (PAID)

The Dutch version of the PAID [20] was used to asses diabetes related distress. For each of its 20 items, patients are requested to indicate how problematic these aspects of diabetes are for them, ranging from 0 (no problem) to 4 (a big problem). The Dutch version of the PAID has been shown to be reliable (Cronbach's alpha.95). Total score is calculated by summation of scores, standardized to a 0–100 range.

#### World Health Organization 5 Well-being Index (WHO-5)

The WHO-5 is a brief measure of well-being that consists of five positively worded items rated on a 6-point scale ranging from 0 (not present) to 5 (constantly present). Total score is calculated by summation of scores, standardized to a 0–100 range. The WHO-5 covers positive mood, vitality, and general interests. The measure has been derived from the WHO-10 and has good psychometric properties [21-24]. Also, both the WHO-10 and WHO-5 have been validated in diabetic patients [25,26].

#### Suffering Question (SQ)

The participants additionally were requested to answer the question 'How much do you suffer from your diabetes?' on a 5-point scale, anchored by not at all (0), and very much (4). This question served to evaluate content validity of the PRISM-RII.

#### Biomedical parameters

Medical data were retrieved from the patient charts. Patients' most recent HbA1c values were documented as an index of glucose control (reflecting the past 6–8 weeks). Values below 7.0% are considered satisfactory. In Addition, the presence of diabetes-related complications (nephropathy, neuropathy, retinopathy, and ulceritis), and comorbidity (e.g. hypertension, rheumatoid arthritis) were documented. For statistical purposes, both variables were scored as 0 (no complications or comorbidity), or 1 (one or more complications or comorbid diseases).

### Statistical analysis

Means and standard deviation of the various self-report measures were calculated. Differences between male and female participants and between type 1 and type 2 diabetes were examined for all measures using Student's t-tests. Mean differences in scores on the PRISM-RII, WHO-5, and PAID between patients with and without complications and/or comorbidity were tested using Students t-tests, as an indication of discriminant validity.

Convergent validity of the PRISM-RII was assessed by calculating mutual Pearson's correlation coefficients between SIS, IPM, PAID and WHO-5. The validity of PRISM-RII for both type 1 and type 2 diabetes was evaluated by computing these correlations for each subtype. Fisher-z transformations of these correlations were used to examine whether these correlations differed significantly from one another.

## Results

Means and standard deviations for all variables are displayed in Table 1.

**Table 1:** Means and standard deviations of SIS, IPM, SQ, WHO-5, PAID, and HbA1c.

	Whole sample N = 308	Type I N = 119	Type II N = 189	t-test for differences
	Mean (SD)	Mean (SD)	Mean (SD)	
SIS	129.6 (99.2)	118.7 (89.2)	136.5 (104.6)	n.s.
IPM	1.8 (0.7)	1.7 (0.7)	1.9 (0.7)	*
SQ	1.7 (1.2)	1.6 (1.1)	1.8 (1.2)	*
WHO-5	64.0 (21.8)	66.6 (16.7)	62.5 (24.4)	(*)
PAID	23.2 (19.5)	19.9 (16.3)	25.2 (21.1)	*
HBA1C	7.9 (1.1)	7.9 (1.1)	7.9 (1.1)	n.s.

SIS = Self-Illness Separation; IPM = Illness Perception Measure; SQ = Suffering Question; WHO-5 = World Health Organisation 5 Well Being Index; PAID = Problem Areas in Diabetes.

n.s. = p > 0.10; (*) 0.05 ≤ p ≤ 0.1, * p ≤ 0.05,

### Type of diabetes

Compared to people with type 1 diabetes, people with type 2 diabetes had a significantly higher IPM score (*t = *-2.34*, p *< .05), and scored significantly higher on the PAID. There were no significant type of diabetes differences for SIS.

### Complications and comorbidity

Patients with complications had a marginally significant higher score on IPM (*t *= -1.76, .05 <*p *< .10), than patients without complications, but SIS did not differ significantly (*t *= -.045, n.s.). Patients with complications had significantly higher scores on the PAID (*t *= -1.97, *p *< .05), and SQ (*t *= -2.96, *p *< .01), and significantly lower scores on the WHO-5 (*t *= 2.01, *p *< .05). Patients with comorbid disorders scored significantly higher on the PAID (*t *= -2.31, *p *< .05), but did not differ significantly on any of the other measures.

### Convergent validity

Correlations between PRISM-RII (IPM and SIS) and other variables are presented in Table 2. The correlation between SIS and IPM was low but significant (*r *= -.23; *p *< .01). As expected, IPM correlated significantly positively with PAID (*r *= .50, *p *< .01) and negatively with WHO-5 (*r *= -.26, *p *< .01). SIS correlated significantly negatively with the PAID (*r *= -.28, *p *< .01), but showed no significant association with WHO-5 (*r *= .08, *p *> .10). Neither IPM nor SIS correlated significantly with HbA1c (*r *= .06, *p *> .10; *r *= .05, *p *> .10, respectively). SIS and IPM both showed, opposite, significant associations with SQ (SIS: *r *= .22, *p *< .01; IPM: *r *= -.36, *p *< .01). Compared to men, women scored significantly higher on the PAID (*t *= -2.00, *p *< .05), and significantly lower on the WHO-5 (*t *= 2.98, *p *< .01). Women also scored higher on the IPM, and this difference approached statistical significance (*t *= -1.85, *p *< .10). There were no significant gender differences for SIS. Comparisons of Fisher-z transformations of type-specific correlations between SIS, IPM, WHO-5, and PAID yielded no significant differences between patient groups.

**Table 2:** Correlations between PRISM-RII measures and SQ, WHO-5, PAID, HbA1c and age.

		IPM	SIS
IPM			-0.25**
SIS		-0.25**	
SQ		0.36**	-0.22**
WHO-5		-0.26**	0.08
PAID		0.50**	-0.28**
HBA1C		0.06	0.05
Age		0.03	0.21**

SIS = Self-Illness Separation; IPM = Illness Perception Measure; SQ = Suffering Question; WHO-5 = World Health Organisation 5 Well Being Index; PAID = Problem Areas in Diabetes.

** p ≤ 0.01

## Discussion

The aim of the present study was to evaluate the validity of the PRIMS-RII as a measure of suffering in people with diabetes.

Although the concept of suffering is abstract, the vast majority of patients was able to complete the PRISM-RII without any difficulty. As for validity, although the PRISM-RII variables did not differentiate between patients with and without complications and comorbidity, both measures were moderately related to diabetes-related distress, as assessed by PAID, while IPM was additionally associated negatively with well-being. In contrast, no association was found between well-being and SIS. This may be related to the more general nature of the well-being construct as measured with the WHO-5, covering issues such as vitality and interest in daily activities. Indeed, there is currently discussion whether generic well-being questionnaires are suitable for use in specific illness populations [27,28]. Another possibility is that the relationship between quality of life and suffering is more complex than assumed. Well-being is a construct emphasizing, in particular, positive emotions, whereas suffering is a construct focussing mainly on negative mood states. This difference might be important, since it has been well established that the presence of negative affect does not necessarily imply the absence of positive affect and vice versa [29]. In that sense, it could be that the positive construct of well-being and the negative construct of suffering might be relatively independent of each other. Alternatively, it has been suggested by Cassell [2] that the ability to give meaning might be a key factor in suffering. More precisely, according to this view a low quality of life is only experienced as suffering if the patient experiences a low quality of life *and *lacks the capacity to give meaning. Future studies should be specifically designed to test this intriguing hypothesis.

We failed to find an association between the PRISM variables and HbA1c as a measure of glycaemic control. This is not unexpected, as HbA1c values do not translate into tangible symptoms, particularly not in patients with reasonably well-controlled diabetes. The fact that the presence of diabetes-related complications and comorbidity was associated with elevated levels of emotional distress but only marginally or not at all with PRISM-variables is counterintuitive. It would suggest that PRISM is less sensitive to diabetes-specific burden than for example the PAID. Moreover, SQ also differed significantly between patients with and without complications, while it was significantly associated with IPM or SIS. More precisely, and as might be expected, a high score on SQ (indicative of more suffering) was related to a lower SIS and a higher IPM. This might be considered as supporting the content validity, i.e. whether PISMR-II is a 'pure' measure of suffering or perhaps also reflects adaptation or coping. Support for this hypothesis further comes from the comments of the patients, who frequently referred to (the success of) their own coping efforts (e.g., "I make sure my diabetes does not become too big an issue in my life"). In a previous study among individuals with problematic alcohol use, Reinhardt et al. [13] also found SIS to discriminate between different levels of readiness to change, which also reflects coping efforts. SIS was partly predicted by age, indicating older people on average having higher SIS scores. This finding suggests more successful adaptation to the disease with increasing age. In additional analysis we controlled for disease duration, showing that this factor per se did not predict SIS, nor did it attenuate the significance of age as a predictor of SIS.

Further research into the content validity of PRISM-RII is warranted, including cognitive debriefing sessions. It is not clear if using a single question on degree of suffering in this context is indeed helpful. First, it should be obvious that the concept of suffering is not easily measured with only one (direct) question. The response to this question hinges entirely on the respondents implicit conceptualisation of suffering, which may differ considerably among patients. Also, suffering may have a strong negative connotation. Perhaps when asked bluntly, suffering is a 'hit or miss' construct, meaning that it is hardly possible to suffer a little bit, as suffering is always associated with serious discomfort. Although responses on the SQ did not approach a binomial distribution, 26% of subjects reported a large amount of suffering, whereas for SIS, 38% of subjects responded in the lowest quarter of possible responses (from 0 to 80), indicative of a large amount of suffering. These differences in distribution also argue in favour of measuring suffering with a visual and indirect method such as the PRISM-RII.

The PRISM has been proven useful in measuring aspects of suffering in several groups of patients, and can be used to compare data across different diseases. However, less is known about how to interpret the relationship between SIS and IPM, which intercorrelate only moderately. SIS is intended to measure the perceived relationship between the patient's self and the illness, which serves as an indicator of suffering. For SIS, a whole or partial overlap of the illness disk with the self may be indicative of a high level of suffering, or, more precisely, strong efforts to cope with living with diabetes. Patients report a variety of explanations in cases of overlap, including "diabetes is a part of me and my life, that is why it overlaps". IPM is intended to measure the patients' perception of the magnitude or severity of the illness. It is tempting to speculate about the correspondence of IPM and SIS with, respectively, problem focused coping (coping efforts to reduce or neutralize the impact of a stressor, in this case the disease) and emotion focused coping (leaving the stressor as it is, but try to better deal with the stressor and its consequences). On the other hand, it could be argued that IPM is more strongly related to PAID and WHO-5, because these latter measures assess the negative impact of diabetes on well-being. Indeed, IPM correlated more strongly with these measures than SIS. Future studies specifically designed to address this issue will yield additional valuable information about the PRISM-RII.

## Conclusion

The PRISM-RII might be a feasible and valid instrument to capture aspects of diabetes-related suffering, unrelated to objective illness characteristics. The PRISM-RII is easily administered, and offers a good starting point for clinical conversations. Future studies should confirm whether PRISM yields additional relevant information to existing measures of psychological well-being and insight into the patients' illness perceptions. The non-verbal PRISM-RII is a promising tool that can be used both in research and clinical settings, in particular also patients with language problems and, maybe, children.

## Competing interests

The authors declare that they have no competing interests.

## Authors' contributions

SK contributed to data collection, performed the statistical analysis and drafted the manuscript. AV designed the computerized PRISM-R measure and contributed to the manuscript.

MdeW contributed to the quality of data management and the manuscript. NZ coordinated the assessments in the diabetes outpatient clinic and data management. FJS conceived of the study, participated in its design and contributed to the manuscript. All authors read and approved the final manuscript.
